# Differentiated service delivery: a qualitative study of people living with HIV and accessing care in a tertiary facility in Ghana

**DOI:** 10.1186/s12913-019-3878-7

**Published:** 2019-02-04

**Authors:** Vincent Adjetey, Dorcas Obiri-Yeboah, Bernard Dornoo

**Affiliations:** 1 Department of Obstetrics and Gynaecology, School of Medical Sciences, Cape Coast, Ghana; 2 Department of Microbiology and Immunology, School of Medical Sciences, College of Health and Allied Sciences, University of Cape Coast, Cape Coast, Ghana; 3 Medical Directorate, University of Professional Studies, Accra, Ghana

**Keywords:** PLHIV, Differentiated Model of Care, Ghana, ART

## Abstract

**Background:**

In 2014, the Joint United Nations Program on HIV/AIDS (UNAIDS) set out a treatment target with the objective to help end the AIDS epidemic by 2030. This was supported by the UNAIDS ’90-90-90’ target that by 2020, 90% of all people living with HIV (PLHIV) will know their HIV status; 90% of all those diagnosed with HIV will be on sustained antiretroviral therapy; and 90% of all people receiving antiretroviral therapy will have viral suppression. The concept of offering differentiated care services using community-based models is evidence-based and is suggested as a means to bring this target into reality. This study sought to explore the possible predictors and acceptability of Community-based health service provision among PLHIV accessing ART services at the Cape Coast Teaching Hospital (CCTH) in Ghana.

**Methods:**

A qualitative study, using 5 focus group discussions (FGD) of 8 participants per group, was conducted at the HIV Clinic in CCTH, in the Central Region of Ghana. Facilitators administered open-ended topic-guided questions. Answers were audio recorded, later transcribed and combined with notes taken during the discussions. Themes around Facility-based and Community-based service delivery and sub-themes developed from the codes, were verified and analyzed by the authors, with the group as the unit for analysis.

**Results:**

Participants expressed preference for facility–based service provision with the construct that, it ensures comprehensive health checks before provision of necessary medications. PLHIV in this study wished that the facility-based visits be more streamlined so “stable clients” could visit twice in a year to reduce the associated time and financial cost. The main barrier to community-based service delivery was concerns about stigmatization and abandonment in the community upon inadvertent disclosure of status.

**Conclusions:**

Although existing evidence suggests that facility-based care was relatively more expensive and time consuming, PLHIV preferred facility-based individualized differentiated model to a community-based model. The fear of stigma and discrimination was very strong and is the main barrier to community-based model among PLHIV in this study and this needs to be explored further and managed.

## Background

At the end of 2015 there were 36.7million people living with HIV globally of which less than half (46%) were on therapy. In Ghana, there were an estimated 313,063 of people living with HIV (PLHIV) at the end of 2017 with 125,667 on antiretroviral therapy (ART) [1]. In 2014, the Joint United Nations Program on HIV/AIDS (UNAIDS) set out an aspiring treatment target with the objective to help end the AIDS epidemic by 2030. This ambitious target was given the needed impetus by the UNAIDS ’90-90-90’ target which was to the effect that by 2020, 90% of all people living with HIV will know their HIV status; 90% of all people with diagnosed HIV infection will receive sustained antiretroviral therapy; 90% of all people receiving antiretroviral therapy will have viral suppression.

There are many challenges affecting access to, and the quality of care for HIV infected clients, including long travelling distances, high transportation costs, long waiting times in health facilities, concerns of confidentiality, issues of stigma and discrimination and poor attitudes of some healthcare workers towards clients. [2]. A lot of work is therefore going into HIV testing and diagnosis, and making therapy easy and accessible for clients.

As part of differentiated care, community health workers, ART clubs and other models of community service delivery are increasingly being used by HIV-treatment programs [3]. It has been recommended that community-based models of ART delivery be used to support ART expansion and retention in resource limited settings [4, 5]. Various models have been proposed and tried in different settings. These include facility-based individual, out –of-facility individual, health care worker managed group and client-managed group models. [4, 6, 7]. For instance, Uganda has the Community Drug Distribution Points (CDDP) for eligible clients (more than 10 weeks on ART, CD4 of more than 350 cells/mm3, and 95% adherence) to pick up their medications near their homes instead of travelling for long distances and queuing for hours [7]. The community-based Adherence Club (CAC) in Cape Town, South Africa is an example of a health care worker managed model. Patients who have been adherent to their treatment regime for more than 12 months and show viral suppression are accepted. They meet about twice a month for “group counselling, brief symptom screen and distribution of prepackaged ART” at an agreed community location [4]. Experience with Mozambique’s Community ART Group (CAGs), an example of a client-managed group, showed that clients felt more empowered and involved in their care and in the creation of a supportive environment leading to improved ART retention. In addition, it led to health services being redirected towards the community and it’s strengthening efforts [2].

Health systems must tailor their anti-retroviral treatment (ART) services such that the client comes first but with due cognizance of their human and financial resource constraints. In Ghana ART service delivery across the country has mainly been facility based per the national guidelines [8]. This approach implies that clients will have to visit the ART clinics in the health facilities regularly in order to receive their medications and necessary follow up care, Currently, Ghana is in the final stages of developing a guideline for differentiated HIV care for the country which aims at improving care and outcomes keeping in mind the ’90 90 90’ targets. Yet, there is limited research evidence from the country on the views of PLHIV in the country regarding their preferred models of care.

Levesque *el at* developed a conceptual framework on access to health care which identifies relevant determinants that impact access from a multilevel perspective [9]. Their revised conceptual framework reveal that factors affecting access to health include those which are related to the health system, institutions, organizations and providers as well as individual, household, community and population level factors. This conceptual framework forms the basis of this study to explore the possible predictors and acceptability of Community-based health service provision among People living with HIV (PLHIV) accessing ART services at the Cape Coast Teaching Hospital in Ghana.

## Methods

### Study design

A qualitative study using focus group discussions (FGD) to explore the notions of clients on community-based delivery of HIV care services as opposed to facility-based care was conducted. Five (5) FGDs were conducted in July 2017 with eight (8) participants in each group.

### Study setting

The study was conducted at the HIV Clinics of the Cape Coast Teaching Hospital (CCTH) situated in the Cape Coast Metropolis of the Central Region of Ghana. Established in 2006, this was the first HIV/ART clinic in the Central Region of Ghana and serves as the referral hospital for the entire region and beyond. The combined HIV care clinics serve an average of 120 clients (children, adolescents and adults) per week.

### Study population

Sampling, and data collection procedure

Focus group participants were randomly selected from the list of clients accessing services at the CCTH, Ghana. The participants were adults, 18 years and above and were grouped according to gender to explore possible gender specific constructs. Persons under 18 years were excluded since they were minors and tend to depend on adult care givers for their clinic visits and health care in general. A trained facilitator led the focus group discussions using the preferred language of the participants (mainly Fante), and a topic guide of open-ended questions. A second trained facilitator documented the group dynamics, general atmosphere, and non-verbal cues of the participants. The FGD guide explored participant’s experiences with ART service delivery so far since their HIV diagnosis. The guide then elicited views on and specific benefits and challenges with the current service deliver. It then explored participant’s understanding of non-facility based ART service delivery and how they perceive the potential benefits and challenges associated with it. Finally the discussion explored their preference of service delivery and why that choice. Written consents were obtained individually prior to FGDs which were held in private locations convenient for the participants, and were audio recorded. After the fifth FGD, it was decided that saturation point had been reached hence no further recruitment was done. Each FGD lasted between 45-60 minutes.

### Data management and analysis

To maintain confidentiality, participants were given codes which were used to identify different voices by the note-taker and used when transcribing and referencing quotations. Each focus group had a unique identifying number written on the focus group discussion forms, in notes taken, and used to name audio files and transcript documents. Audio recordings were transcribed into Word, and stored on a password protected laptop. The transcription was proof-read against the audio file by both the transcriber and a supervising member of the research team to check for accuracy, identify any missed or misheard words and clarify any areas of confusion or unclear terminology. Notes were translated into English from local languages where required.

Indexed coding was used by the authors to analyze data from the FGDs. After transcription of FGDs and familiarization with the transcripts and associated notes, transcripts were coded line by line independently by the two FGD facilitators. There was no significant conflict with codes and minor differences were resolved by one researcher. From the codes, various themes emerged under Facility-based service and Community-based service delivery models and these themes were developed, verified and analyzed with the group as the unit for analysis. Sub-themes which emerged were further analyzed and developed. Under facility-based service, the subtheme descriptors included opportunity for comprehensive care, the benefits and challenges associated with distance to clinics, increase interval between clinic visits and efficient clinic organization to reduce wait times. Sub-theme descriptors for community-based services were the convenience of “home” service delivery, inadvertent disclosure with resultant stigma and discrimination, and challenges with organization of community-based service. These are presented in the results section and then discussed.

### Ethical issues

This study was approved by the Institutional Review Board of the University of Cape-Coast (UCCIRB /EXT/2017/03), Ghana. Permission to undertake the study at the HIV Clinics of the hospital was sought and granted by the hospital management. The participants enrolled in the study gave written informed consent after full explanation of the procedure in the language and/or dialect they best understood. None of the authors were present during the FGDs so as to reduce desirability bias and any discomfort to the participants.

## Results

### Participants’ background

There were 16 (2 FGDs) males and 24 females (3 FGDs) in total. All males had at least a primary level education whereas a total of 6 females had no formal education at all. Table 1 gives the details of the participants in each group.

**Table 1.** Characteristics of the 8 participants making up each FGD

Variable	FGD 1 (^a^M)	FGD 2 (^b^F)	FGD 3 (F)	FGD 4 (M)	FGD 5 (F)
Age (years)					
Mean (SD)	46.4 (11.3)	52.1 (14.0)	46.3 (7.1)	48.5 (11.0)	49.3 (12.3)
Range	32, 60	35,79	37, 59	29, 62	24, 62
Educational level, n (%)					
None	0 (0.0)	3 (37.5)	2 (25.0)	0 (0.0)	1 (12.5)
≤6 years of education	3 (37.5)	2 (25.0)	3 (37.5)	2 (25.0)	3 (37.5)
JHS/form 4 level	3 (37.5)	3 (37.5)	1 (12.5)	3 (37.5)	4 (50.0)
SHS	2 (25.0)	0 (0.0)	1 (12.5)	2 (25.0)	0 (0.0)
Tertiary	0 (0.0)	0 (0.0)	1 (12.5)	1 (12.5)	0 (0.0)
Employment, n (%)					
Unemployed	2 (25.0)	3 (37.5)	3 (37.5)	0 (0.0)	3 (37.5)
Unskilled employment	6 (75.0)	5 (62.5)	4 (50.0)	7 87.5)	5 (62.5)
Skilled	0 (0.0)	0 (0.0)	1 (12.5)	1 (12.5)	0 (0.0)
Duration on ART (years)					
<1	0 (0.0)	0 (0.0)	0 (0.0)	0 (0.0)	0 (0.0)
1 – <5	5 (62.5)	3 (37.5)	7 87.5)	1 (12.5)	3 (37.5)
5 - <10	3 (37.5)	1 (12.5)	1 (12.5)	6 (75.0)	4 (50.0)
≥10	0 (0.0)	4 (50.0)	0 (0.0)	1 (12.5)	1 (12.5)

^a^M= Male ^b^F= Female

### Facility-Based Service Delivery

#### Opportunity for comprehensive care

Participants generally felt that regular (1-2 monthly) attendance at facilities for medical reviews with clinicians, possible laboratory tests and medication refills had advantages. They associated a clean bill of health from providers to a better life as exemplified in the following narratives:

*“Coming here, I get to see the doctor, go to the lab and get to do other things in the hospital that is why I like coming here.”* (Female, 7 years on ART, FGD2)

*“When we come to the hospital, it helps because they talk to us on various things to make sure we take the drugs. Different healthy meals are also talked about, and other things for a good life. I believe coming to clinic has a psychological effect also because the moment you meet a doctor you start to feel better. So I prefer coming over to see the doctor every time”.* (Male, 7 years on ART, FGD4)

***“****All I want is to come* (to the health facility). *Maybe there will be something that needs to be checked in my system before the doctor gives more medication. If the drugs are brought to me at home by someone that cannot be detected”.* (Male, >10 years on ART, FGD1)

#### The benefit of distance to the clinic

Many in the groups felt that, coming to a health facility far away from their communities was actually a way of maintaining anonymity and avoiding unintended disclosure of their HIV status, and the associated stigma and discrimination in their communities as below.

*“I come to this hospital to avoid people I know seeing me. I could have gone to the Saltpond hospital* (closer to participant) *but I come all the way here. After all who ever will see me in this clinic also has the HIV”.* (Female, 8 years on ART, FGD5)

#### Challenges with distance to the clinic

Despite the construct of the distance to the clinic being beneficial, some participants also saw it as a challenge due to the cost implication and effect on honouring clinic appointment. *“Transportation is very costly for me. My child and I spent ₵160 ($40) today when coming and that is my problem. It make it difficult for me and sometime I cannot come.”* (Male, 22 years on ART, FGD1)

#### Increase interval between clinic visits

Participants in this study also presented constructs of the challenges associated with the facility based service provision which impacts them negatively. In their constructs, they offered possible solutions which in their opinion would improve the care of PLHIV:

*“Now they give many of us 1 month or two. We wish they give us longer time to come back”.* (Males, all participants, FGD1)

“*I prefer coming but would want to take the medication for like 4 to 5 months. When I’m due for the next set, then I come for my drugs”.* (Female, 3 years on ART, FGD2)

#### Efficient clinic organization to reduce waiting times

The need to improve the organization and efficiency of service delivery during clinic visits were also highlighted. As can be seen from quotes below, participants felt that they spent too much time at the clinic and some offered possible interventions to reduce the time for all clients.

*“As she is saying, then the folders should be arranged well. This is because you cannot come and sit here from dawn and waste your whole time. People come on empty stomachs and come from far so the folders need to be arranged well. So the queue should be arranged well”.* (Female, 8 years on ART, FGD5)

*“Again, the time we spend here is too much here. We have to come and go back to our jobs. We come very early but leave quite late.”* (Male, 7 years on ART, FGD 1)

#### Community-based service delivery

Among these study groups of PLHIV, the concept of community based ART delivery and follow up was met with very strong opinions. While the concept in general was acceptable to some, no group had a consensus.

#### The convenience of “home” service delivery

For participants who indicated their preference for community-based service delivery, the focus seemed to be on reduction in the cost of travelling and convenience of accessing medications at the comfort of their own home:

*“I live in Takoradi* (1 hour drive away) *but come all the way here to take my medication. This is because I do not want people I know to find out. I will not want to visit any hospital close by to take my drugs, but if they can bring them to our homes, then that will be fine”.* (Female, about 2 years on ART, FGD3)

*“I believe that in advanced countries, you can order your drugs even when you are home so if I’m in my community and my health care practitioner brings my drugs it is rather to my advantage. So if my drugs are brought to me in my comfort zone, I will be glad”.* (Male, 9 years on ART, FGD4)

#### Inadvertent disclosure with resultant stigma and discrimination

Participants from all 5 FGDs expressed strong feelings about any service provided in their community on the basis of fear of accidental disclosure and its associated stigma and discrimination. The general construct was of the opinion that such an approach will require visits from health care workers and others to their homes. They felt that, it will be almost impossible to arrange such community-based strategies without people becoming suspicious:

*“We do not want people to know that we have this condition. It is a private issue. If they start coming that often to our houses, people will start asking questions.”* (Female, 1 year on ART, FGD3)

*“I do not like that idea* (community-based service*) I prefer to come here* (the health facility)*. People will start getting suspicious when we meet at a particular spot in our communities.”* (Male, 2 years on ART, FGD1)

*“The nurses in my community will be the very ones to broadcast my disease to people. Some of the nurses talk too much.”* (Female, 10 years on ART, FGD2)

#### Doubts regarding the organization of community-based service delivery

Participants were unsure about how such a model will be organized. One participant felt that this service would depend on having a significant number of PLHIV in a particular community. Such numbers would have allowed a peer-led approach but the current situation, he felt, might make it impractical to organize across the country:

*“If we were to be about 10 or 15 then it would have been better to group ourselves and meet someone at a particular place for our drugs. We really wish to have the drugs brought to us but we cannot force if we are very few or even just one person in a particular place”.* (Male, 2 years on ART, FGD3)

Uncertainty about how this would be organized, led to constructs about how this could cause problems rather for PLHIV as seen below:

*“How about when the doctor comes and meets your absence. He or she will definitely leave the drugs and that will obviously give you the patient up. So I really don’t support the drugs being brought to our communities.”* (Male, 6 years on ART, FGD4)

## Discussion

Differentiated HIV care models among other reasons aim at taking care close to clients to improve access while also decongesting health facilities [7]. Frequent attendance to the hospital facility is said to be expensive for these often-poor patients and that for the “90-90-90” targets to be achieved differentiated service provision is essential. To provide the best acceptable service model for clients in specific settings is paramount to achieving the desired outcome [4, 7].

In this study participants were apprehensive of the idea of bringing care closer to their door-steps mainly because of the fear of stigmatization and the resultant discrimination. Thus most participants seemed to propose and indicate a preference for the facility-based individual model with specifics about prolonged intervals (more than 3 months) between medical reviews and/or refills. They felt that this will reduce the rates of default on clinic appointment. This, could likely improve overall outcome for PLHIV as found in similar settings in countries such as South Africa [4]. Further, job and routines of the PLHIV will not be disrupted as much hence improving their quality of life. In their own narratives they recognize that this will be more appropriate for clients who are “stable” on their medications. This is in line with what is proposed in the ART guidelines for PLHIV in Ghana [10] and will be beneficial if all ART sites implemented this. In addition, participants felt that they spent too much time at the facility when they attended their appointments. Some just needed medication refills. Others need reviews with/without laboratory work. Streamlining the process such that those needing only refills can go straight to the pharmacist to review what is needed and refill their medications, will be helpful albeit going against those who see the facility-based care as an opportunity for comprehensive health check.

A point of significant interest was the fact that most of the FGD participants preferred to receive their care at the hospital facility even if the government paid for the cost of bringing care into the community, and closer to their homes. In a sense it demonstrates the confidence participants have in those caring for them in the facilities, and their own motivation to have ART. On the other hand, it may be a point to how strong stigma and discrimination about HIV status influences access to treatment as reported in other studies too [11, 12]. In other words, because of the fear of stigmatization, patients would rather travel long distances at relatively significant expense than have care brought to them at/or close to home. Many participants claimed *“I do not want anyone coming. I will come”* (to the hospital). This area of stigma needs to be studies further and in specific populations to identify areas for targeted interventions. A study in the United State looked at culturally appropriate scales for measuring stigma among African-Americans living with HIV and found such a scale useful [13].

Privacy and preventing others from pointing fingers at them were of paramount concern. Those few who preferred care close to home suggested providers could bring the medications to community-based health providers, like community health nurses where they can meet providers for review and collect their refills. Their reasoning was similar to the Out-of-facility individual model, and the health care worker managed group model which includes clinical consultation [6, 7, 14]. The idea of having a clinical consultation could explain why some participants in this study preferred hospital based care, because it addresses their preference for health worker assessment of their health at each visit before they collect their medications. This concern though may be imagined than real as a number of studies have reported that health outcomes are similar among clients in community-based and facility-based ART care delivery models [15, 16]. Most groups had the construct that, this will ensure that “nothing is missed” in their health status. This clearly shows that any community-based service would have to be organized to ensure that some basic health check, such as tuberculosis symptoms screening, can be carried out for clients if this model is practiced in the setting of these participants [3]. Such assurance and arrangement will increase confidence among clients and reduce losses to follow up, as found in other studies [5]. In addition community-based organizations have an important role to play and their presence in communities could be harnessed to improve access to a range of services including ART provision if the needed training and supervisory support are given [17].

Some FGD comments bring the need for patient confidentiality into sharp focus. For a participant to state that “some of the nurses talk too much” and “the nurses in my community will be the very ones to broadcast a person’s status”, attests to the gravity of the concern. This comment touches on a broader issue of professional practice and the ethics of nursing and health care delivery, which are important but not a subject of discussion in this paper. In addition, one participant sadly stated thus: “*when people get to know they’ll abandon me*”. Education of health care workers and interventions in communities will be essential in reducing this barrier to care in the Ghanaian context as seen in various studies [18–21].

Gender has been found to influence the all-cause mortality among PLHIV in low and middle income countries (LMIC) including evidence from a systematic review [22] hence the need to study factors which will positively affect retention in care and adherence among males and females [23–25]. In this study, males had overall higher formal educational level and better employment situation, factors which can affect the response to ART among clients [26]. Females in these discussions generally focused on any model which protects their privacy and prevented people in their communities from becoming aware of their status including fears associated with the potential negative effects of accidental disclosure to their sexual partners as found in another study on disclosure [27]. The males while also having concerns about confidentiality in these discussions, had a strong preference for facility-based service delivery as long as waiting time at clinics are reduced and review intervals are lengthened.

These various components and factors which affect choice of HIV positive clients concerning the service delivery model are discussed by Barker *et al* in their study which estimated the cost of various service delivery models. Their differentiated care conceptual framework looked at various components which affect the service delivery model including frequency and type of clinic visits, laboratory testing needs and the community-based ART [28]. The framework by Levesque *et al* suggested five dimensions to accessibility (approachability; acceptability; availability and accommodation; affordability; and appropriateness) and five corresponding abilities of populations (ability to perceive; ability to seek; ability to reach; ability to pay; and ability to engage) [9]. Finding from this study supports similar factors which influenced participant’s perspective on facility-based and community-based service delivery in this study.

Limitations

Not all people are comfortable discussing their health issues in a group. Individual in-depth interviews could have revealed other hidden concerns that affected participant’s opinion of facility based and community based health service delivery. In addition, the study was limited to a single tertiary facility in Ghana. Inclusion of facilities from other levels of healthcare may have enhance the applicability of the findings to a broader context.

## Conclusion

Although existing evidence suggests that facility-based care was relatively more expensive and time consuming, PLHIV in this study preferred facility-based individualized differentiated model to a community-based model. The fear of stigma and discrimination was very strong and was the main barrier to community-based model. There is a need for further studies in various settings which must be designed to provide better and relevant perspectives on group-based and other differentiated models, and explore what would help to improve their implementation [2, 6]. Such studies will inform policy makers on the best approach to differentiated service provision in their specific context [29]. It may highlight other issues apart from cost and time consumption in attending clinic appointments.

## ABBREVIATIONS

AIDS: Acquired immunodeficiency syndrome
ART: Antiretroviral therapy
CCTH: Cape coast teaching hospital
FGD: Focus group discussion
HIV: Human immunodeficiency virus
IRB: Institutional review board
NACP: National AIDS control programme
PLHIV: People living with HIV
UCC: University of Cape Coast
UNAIDS: The joint united nations program on HIV/AIDS
VL: Viral load
WHO: World health organization
